# Xenogeneic modulation of the ClpCP protease of *Bacillus subtilis* by a phage-encoded adaptor-like protein

**DOI:** 10.1074/jbc.RA119.010007

**Published:** 2019-07-30

**Authors:** Nancy Mulvenna, Ingo Hantke, Lynn Burchell, Sophie Nicod, David Bell, Kürşad Turgay, Sivaramesh Wigneshweraraj

**Affiliations:** ‡ MRC Centre for Molecular Bacteriology and Infection, Imperial College London, London SW7 2AZ, United Kingdom; § Institute für Mikrobiologie, Leibniz Universität Hannover, Herrenhäuser Str. 2, 30419 Hannover, Germany; ¶ SynbiCITE, iHub, Imperial College London, White City, London W12 0BZ, United Kingdom; ‖ Max Planck Unit for the Science of Pathogens, Chariteplatz 1, 10117 Berlin, Germany

**Keywords:** bacteriophage, bacteria, ATP-dependent protease, chaperone, Bacillus

## Abstract

Like eukaryotic and archaeal viruses, which coopt the host's cellular pathways for their replication, bacteriophages have evolved strategies to alter the metabolism of their bacterial host. SPO1 bacteriophage infection of *Bacillus subtilis* results in comprehensive remodeling of cellular processes, leading to conversion of the bacterial cell into a factory for phage progeny production. A cluster of 26 genes in the SPO1 genome, called the host takeover module, encodes for potentially cytotoxic proteins that specifically shut down various processes in the bacterial host, including transcription, DNA synthesis, and cell division. However, the properties and bacterial targets of many genes of the SPO1 host takeover module remain elusive. Through a systematic analysis of gene products encoded by the SPO1 host takeover module, here we identified eight gene products that attenuated *B. subtilis* growth. Of the eight phage gene products that attenuated bacterial growth, a 25-kDa protein called Gp53 was shown to interact with the AAA+ chaperone protein ClpC of the ClpCP protease of *B. subtilis*. Our results further reveal that Gp53 is a phage-encoded adaptor-like protein that modulates the activity of the ClpCP protease to enable efficient SPO1 phage progeny development. In summary, our findings indicate that the bacterial ClpCP protease is the target of xenogeneic (dys)regulation by a SPO1 phage–derived factor and add Gp53 to the list of antibacterial products that target bacterial protein degradation and therefore may have utility for the development of novel antibacterial agents.

## Introduction

**Figure 1.**
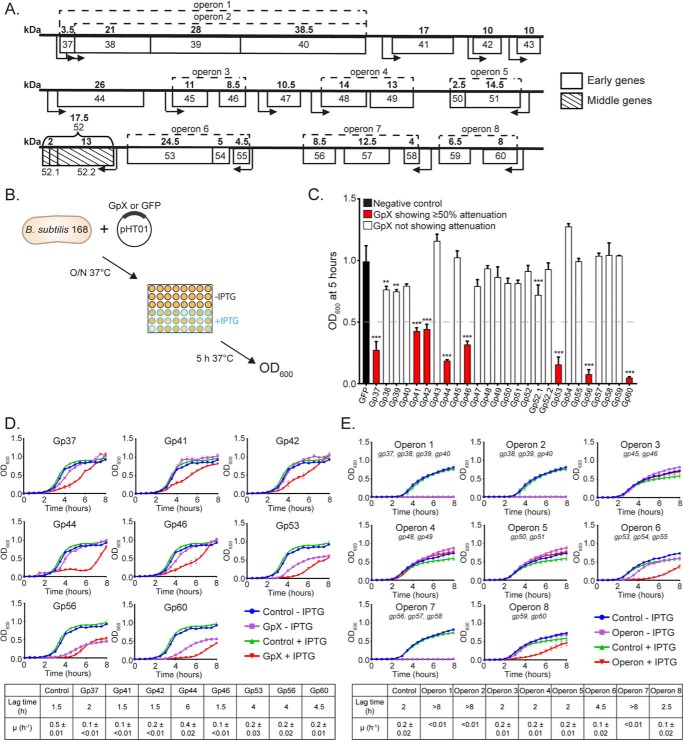
**SPO1 host takeover module genes that attenuate *B. subtilis* growth.** *A*, schematic of the SPO1 host takeover module. The molecular masses (kilodaltons) of the individual gene products are shown above each gene in *bold*, and operons are indicated by *dotted lines*. The predicted positions of promoters are shown as *arrows*, indicating the direction of transcription. *B*, schematic of the experimental procedure used to identify SPO1 host takeover module gene products that attenuate the growth of *B. subtilis. O/N*, overnight. *C*, graph showing the *A*_600_ values of *B. subtilis* cultures at 5 h of growth in the presence of IPTG, which induces expression of the individual host takeover module genes. Gene products shown in *red* displayed 50% or more attenuation compared with control cells expressing GFP. *D*, graphs showing growth curves (*red*) of *B. subtilis* cultures expressing SPO1 host takeover module genes that attenuated growth 50% or more and that of control cultures (see *key*). *E*, graphs showing growth curves (*red*) of *B. subtilis* cultures expressing the individual operons of the SPO1 host takeover module and that of control cultures (see *key*). The lag time preceding growth and growth rate (μ) of *B. subtilis* cultures expressing SPO1 host takeover module gene product(s) is shown in the *bottom panels* in *D* and *E. Error bars* in *C–E* represent S.E. (*n* = 3). Statistical analyses were performed by one-way ANOVA (**, *p* < 0.01; ***, *p* < 0.001).

Much like eukaryotic and archaeal viruses, which derail the host's cellular processes to facilitate viral replication, phages have evolved complex strategies to acquire their bacterial hosts. To successfully infect and replicate in the bacterial cell, many phages encode proteins that specifically interfere with essential biological processes of the host bacterium, including transcription, translation, DNA replication, and cell division (1). Phage proteins that interfere with host processes are typically small in size (on average ∼160 amino acid residues) and are usually produced at high levels early in the infection cycle (2). SPO1 is a prototypical lytic phage of *Bacillus subtilis*, and its genes are categorized as early, middle, and late to reflect the time of their expression during SPO1 infection in *B. subtilis*. The majority of SPO1 early genes associated with host takeover are in the 12.4-kb terminal region of the genome, which includes the 26-gene host takeover module (Fig. 1*A*) (3, 4). The genes within the host takeover module, genes *37–60*, have several hallmarks to suit the characteristics of phage proteins that interfere with host processes: they are mostly small and produced early in infection and contain promoters and ribosome binding sites characteristic of highly expressed genes (3, 5). Many of them have been shown previously to be involved in the shutoff of bacterial DNA and RNA synthesis (*gp38*, *gp39*, *gp40*, *gp44*, *gp50*, and *gp51*) or to inhibit cell division (*gp56*) during SPO1 infection (6–8). Further, plasmid-borne expression of *gp44*, *gp56,* and *gps50/51* in *B. subtilis* has been shown to attenuate growth and reduce viability, respectively (6, 8, 9). With the exception of the product of *gp44*, which has been postulated to interact with *B. subtilis* RNA polymerase (9, 10), the bacterial targets and mechanism of action of the gene products encoded by the host takeover module of SPO1 remain elusive. Clearly, phages and their gene products represent an underexploited resource for potentially developing novel antibacterial strategies and to gain new insights into bacterial cell function and regulation. In this study, we undertook a systematic approach to identify genes in the SPO1 phage host takeover module that had a detrimental effect on *B. subtilis* growth and unveil the biological role of the product of *gp53*, which interacts with the Hsp100/Clp family member ClpC of *B. subtilis*.

## Results

### The effect of SPO1 host takeover module genes on B. subtilis growth

We wanted to identify genes in the SPO1 host takeover module that had a detrimental effect on *B. subtilis* growth by growing bacteria in the absence and presence of isopropyl 1-thio-β-d-galactopyranoside (IPTG),^3^ which allowed plasmid-borne (pHT01 (11)) expression of the 26 host takeover genes either individually or with other genes in their respective operons (Fig. 1*B*). Any effect of the gene products of the host takeover module on *B. subtilis* growth was monitored by determining the cell density by measuring light absorbance of the culture at 600 nm after a 5-h period of incubation at 37 °C (Fig. 1*B*). As the control, we used bacteria containing the pHT01 plasmid expressing GFP. As shown in Fig. 1*C*, when the SPO1 phage host takeover module genes were expressed individually in *B. subtilis*, the growth of bacteria expressing Gp37, Gp41, Gp42, Gp44, Gp46, Gp53, Gp56, and Gp60 was attenuated by 50% or more compared with control cells expressing GFP. The individual graphs in Fig. 1*D* show growth curves of *B. subtilis* expressing Gp37, Gp41, Gp42, Gp44, Gp46, Gp53, Gp56, and Gp60 over a period of 8 h. We noted that, under our conditions, plasmid-borne expression of Gp37, Gp41, Gp42, Gp44, Gp46, Gp53, Gp56, and Gp60 slowed the growth rate (μ) to varying degrees but, in the cases of Gp37, Gp44, Gp53, Gp56, and Gp60, also attenuated growth by extending the lag time preceding growth (Fig. 1*D*). Further, it seemed that leaky expression (which occurs in the absence of the inducer) of Gp53, Gp56, and Gp60 also attenuated growth to some degree, indicating that the latter SPO1 gene products are potentially more toxic to *B. subtilis* than the others (*i.e.* Gp37, Gp41, Gp42, Gp44, and Gp46). The expression of the SPO1 host takeover module genes together with other genes in their respective operons revealed that operons containing genes shown to attenuate growth when expressed individually, as expected, attenuated growth efficiently (Fig. 1*E*) with the following exceptions. First, Gp38, Gp39, and Gp40, when expressed together in operon 1 and operon 2, appeared to act synergistically and displayed an enhanced ability to attenuate bacterial growth (compare Fig. 1, *C* and *E*). Further, we note that, in *B. subtilis*, cells in which the host takeover module genes in operon 1 and 2 as well as 7 are expressed together do not recover growth under our experimental conditions, as observed when the genes are expressed individually (compare Fig. 1, *D* and *E*). This indicates that the host takeover module gene products within each operon functionally interact and thus have a more pronounced effect on host physiology than when expressed individually. Second, we note that Gp46 is no longer able to attenuate growth of *B. subtilis* when expressed together with Gp45 in operon 3. This implies that Gp45 somehow mitigates the antagonistic effect of Gp46 on *B. subtilis* cells. Overall, we conclude that recombinant forms of Gp37, Gp41, Gp42, Gp44, Gp46, Gp53, Gp56, and Gp60 have a detrimental effect on *B. subtilis* growth in the absence of SPO1 infection, presumably by targeting essential cellular processes.

### Gp53 interacts with the ClpC ATPase of the ClpCP protease in B. subtilis

**Figure 2.**
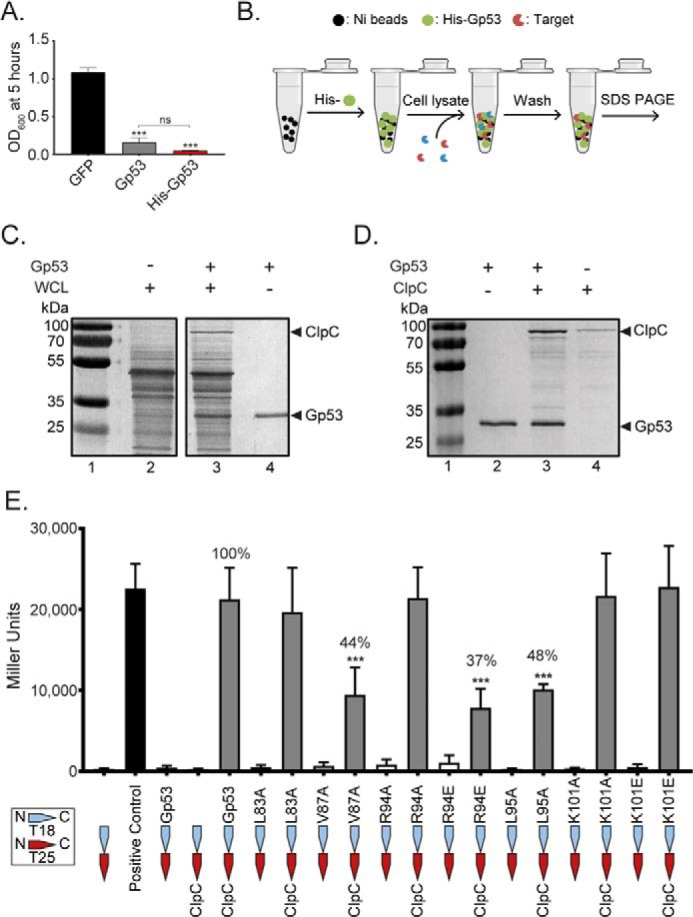
**Gp53 interacts with the ClpC ATPase of the ClpCP protease in *B. subtilis*.** *A*, bar chart comparing the efficacy of growth attenuation of a culture of *B. subtilis* either expressing N-terminal His_6_-tagged Gp53 (*red*) or untagged Gp53 (*gray*). *B*, schematic of the pulldown assay used to identify the bacterial target(s) of Gp53. *C*, a representative image of an SDS-PAGE gel showing results of the pulldown assay with Gp53 and whole-cell extracts (*WCL*) of *B. subtilis.* The band specifically enriched in reactions containing immobilized Gp53 is indicated by an *arrowhead* in *lane 3. D*, a representative image of an SDS-PAGE gel showing results of the pulldown assay with purified Gp53 and N-terminal FLAG-tagged ClpC. The migration positions of Gp53 and ClpC are indicated. *E*, bar chart showing the results from the bacterial two-hybrid interaction assay with ClpC and mutant variants of Gp53. The ClpC-binding activity of the Gp53 mutants as a percentage of WT Gp53 activity is indicated. *Error bars* in *A* and *E* represent S.E. (*n* = 3). Statistical analyses were performed by one-way ANOVA (*ns*, not significant; ***, *p* < 0.001).

Because Gp53 was experimentally more tractable than the other SPO1 host takeover factor gene products, we next focused on identifying the target(s) of Gp53 in *B. subtilis*. We constructed an N-terminal His_6_-tagged version of Gp53 to identify its bacterial target(s) by conducting a pulldown assay using whole-cell extracts of exponentially growing *B. subtilis* cells. Initially, we investigated whether the histidine-tagged version of Gp53 retained its ability to attenuate *B. subtilis* growth under the conditions described in Fig. 1*C*. As shown in Fig. 2*A*, the activity of N-terminal His_6_-tagged Gp53 and its untagged counterpart did not differ significantly. For simplicity, from here on, the N-terminal His_6_-tagged version of Gp53 will be referred to as Gp53. To perform the pulldown assays, purified Gp53 was immobilized onto nickel resin, and the “charged” resin was incubated with whole-cell extracts prepared from exponentially growing *B. subtilis* cells (Fig. 2*B*). The resin was then washed extensively to remove any nonspecific interactions before analysis by SDS-PAGE. As shown in Fig. 2*C*, when the pulldown assay was conducted in the presence of Gp53, we detected specific enrichment of a band on the SDS-PAGE gel (Fig. 2*C*, *arrowhead*, *lane 3*), which was not observed in the control reactions with “uncharged” resin (*i.e.* in the absence of any immobilized protein) (Fig. 2*C*, *lane 2*). The enriched band was investigated by linear quadrupole ion trap Fourier transform MS analysis, which revealed it to be the Hsp100/Clp family member ClpC, the ATPase subunit of the ClpCP protease in *B. subtilis*. To further validate that Gp53 interacts with ClpC, we repeated the pulldown assay using purified C-terminal FLAG-tagged ClpC and nickel resin with immobilized Gp53. As shown in Fig. 2*D*, FLAG-tagged ClpC appears to weakly interact with the nickel resin (*lane 4*) in the absence of Gp53. However, a specific enrichment of ClpC is clearly seen in the presence of Gp53 (Fig. 2*D*, *lane 3*).

To establish that the interaction between Gp53 and ClpC is specific and to identify amino acids in Gp53 important for binding to ClpC, we conducted a BLAST search using standard search parameters and SPO1 Gp53 as a query sequence. Three homologous proteins and one protein fragment from SPO1-related phages were found (Fig. S1), with amino acids (Leu-83, Val-87, Arg-94, Leu-95, and Lys-101) conserved across all five sequences. All of these residues were individually substituted with alanine, apart from the positively charged residues Arg-94 and Lys-101, which were also replaced with negatively charged glutamic acid residues. Next, a bacterial two-hybrid (BTH) interaction assay was performed to determine how the amino acid substitutions in Gp53 affected its ability to interact with ClpC. We opted for the bacterial adenylate cyclase two-hybrid system, in which both *gp53* and *clpC* were coexpressed in a Δ*cya Escherichia coli* strain DHM1 as fusions to one of two fragments (T18 and T25) of the catalytic domain of *Bordetella pertussis* adenylate cyclase (12). Interaction of two-hybrid proteins results in a functional complementation between T18 and T25, leading to cAMP synthesis, and, consequently, transcriptional activation of the lactose operon that can be detected in a β-gal assay. As shown in Fig. 2*E*, reactions with Gp53 variants harboring an alanine substitution (V87A or L95A) and charge-reversal substitution at Arg-94 (R94E) displayed significantly lower β-gal activity compared with the reaction with WT Gp53. We conclude that the proximally located amino acid residues Val-87, Arg-94, and Leu-95 in Gp53 are important determinants for binding to ClpC.

### Gp53 stimulates the ATPase activity of ClpC in a manner analogous to B. subtilis adaptor proteins

**Figure 3.**
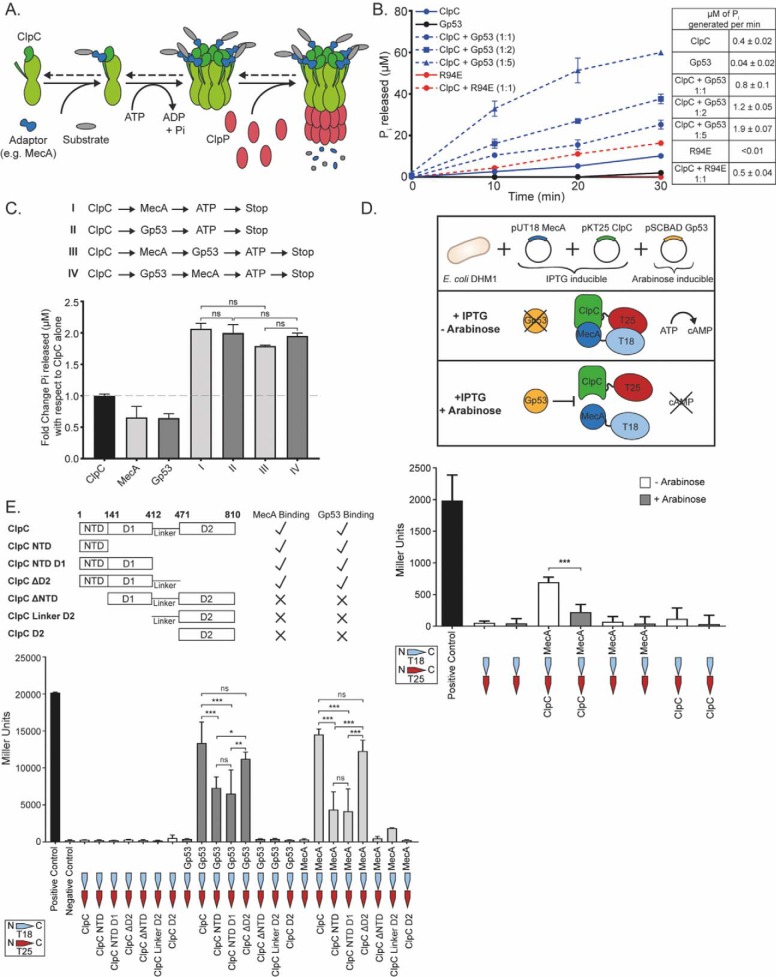
**Gp53 stimulates the ATPase activity of ClpC and competes with the *B. subtilis* adaptor protein MecA for binding to ClpC.** *A*, schematic showing how the ATP hydrolysis and adaptor protein mediated formation of the functional ClpCP protease in *B. subtilis* (adapted from Molière *et al.* (32)). *B*, graph showing the amount of ATP hydrolyzed (P_i_ release, micromolar) as a function of time by ClpC (0.2 μm) alone and in the presence of different amounts of Gp53 (0.2, 0.4, and 1 μm). Numerical ATPase rates are shown on the *right. C*, bar chart showing results from the ATPase assay (as in *B*) in which ClpC (50 nm) was incubated with equimolar amounts of MecA (*reaction I*), Gp53 (*reaction II*), or MecA and Gp53 (added to the reaction in different orders, *reactions III* and *IV*). The amount of P_i_ released (micromolar) is expressed as -fold change with respect to the reaction with ClpC alone, *i.e.* its basal ATPase activity. *D*, *bottom panel*, bar chart showing the results from the modified bacterial two-hybrid interaction assay to demonstrate that Gp53 competes with MecA for binding ClpC. *Top panel*, the assay setup (see text for details). *E*, *bottom panel*, bar chart showing the results from the bacterial two-hybrid assay demonstrating binding of Gp53 or MecA to different domains of ClpC (as shown in the *top panel*). In *B–E*, *error bars* represent S.E. (*n* = 3). Statistical analyses were performed by one-way ANOVA (*ns*, not significant; **, *p* < 0.01; *** *p* < 0.001).

A major role of ClpC in *B. subtilis* is ATP hydrolysis-dependent unfolding and loading of substrate proteins for degradation by the protease ClpP. Substrate specificity upon ClpC is conferred by different adaptor proteins that interact with ClpC and trigger oligomerization, thereby allowing subsequent formation of a complex with ClpP monomers that come together to form the proteolytic chamber (Fig. 3*A*). In other words, the adaptor protein is an obligatory activator of the ClpCP protease (13). Because binding of the adaptor protein, such as the well-documented MecA protein, has been shown to stimulate the basal ATPase activity of ClpC, we initially tested how Gp53 binding affected the ATPase activity of ClpC. The results shown in Fig. 3*B* indicate dose-dependent stimulation of the ATPase activity of ClpC by Gp53. Control experiments with the mutant variant of Gp53 harboring the R94E substitution, which displayed compromised ability to bind ClpC in the BTH assay (Fig. 2*E*), revealed that the stimulation of ClpC's basal ATPase activity was due to the specific action of Gp53 (Fig. 3*B*). We next wanted to determine whether Gp53 competed with native adaptor proteins for binding to ClpC. Using MecA as a model adaptor protein (14–16), we initially conducted ATPase assays to determine whether Gp53 and MecA can bind simultaneously to ClpC and can act synergistically to stimulate the basal ATPase activity of ClpC. As shown in Fig. 3*C*, addition of MecA (*reaction I*) or Gp53 (*reaction II*) resulted in stimulation of the basal ATPase activity of ClpC. However, the presence of MecA and Gp53 together in the reaction, regardless of the order of addition, did not result in an increase in ClpC's ATPase activity to a level higher than the ATPase activity seen when MecA and Gp53 were added individually (Fig. 3*C*, compare *reactions I* and *II* with *III* and *IV*). Therefore, we conclude that MecA and Gp53 do not synergistically stimulate the ATPase activity of ClpC. However, because MecA and Gp53 individually stimulate the basal ATPase activity of ClpC to comparable levels (Fig. 3*C*, *reactions I* and *II*), it is not possible to tell whether they are competing for ClpC binding in the context of this assay. Therefore, to directly determine that Gp53 competes with MecA for binding to ClpC, we used a modified version of the BTH assay described in Fig. 2*E*. In this assay, MecA and ClpC were fused to the T18 and T25 fragments, respectively, of the catalytic domain of *B. pertussis* adenylate cyclase and transformed into Δ*cya E. coli* strain DHM1 containing a plasmid in which Gp53 expression was under the control of the l-arabinose–inducible *araB* promoter. We expected that if Gp53 competed with MecA for binding to ClpC, then the productive interaction between MecA and ClpC would be disrupted when expression of Gp53 is induced with l-arabinose (Fig. 3*D*, *schematic*). As expected, the results revealed that the β-gal activity originating from the productive interaction between MecA and ClpC was reduced by ∼3-fold in the presence of l-arabinose (Fig. 3*D*). Additional control BTH assays revealed that Gp53 and MecA do not interact (Fig. S2), suggesting that the reduction in β-gal activity originating from the productive interaction between MecA and ClpC in the presence of Gp53 (Fig. 3*D*) was not due to MecA being titrated away from ClpC by Gp53. Further, previous studies (17–20) revealed the N-terminal domain (amino acid residues 1–141) and a linker region (amino acid residues 412–471) in ClpC to be important for binding to the adaptor proteins MecA and McsB. Therefore, to determine whether the Gp53- and MecA/McsB-interacting regions on ClpC overlap or are different, we fused six fragments of ClpC (Fig. 3*E*, *schematic*) to T25 and used either MecA or Gp53 fused to T18 in the BTH assay. The results shown in Fig. 3*E* clearly reveal that both MecA and Gp53 bind to overlapping surfaces on ClpC, with the N-terminal domain of ClpC accounting for much of the binding and the linker region restoring the interaction to that seen with full-length ClpC. In summary, we conclude that Gp53, although it clearly stimulates the ATPase activity of ClpC in a manner analogous to *B. subtilis* adaptor proteins, is likely to compete with the latter for binding to ClpC. Thus, by inference, we suggest that Gp53 could affect the normal functioning of the ClpCP protease by excluding the functionally obligatory adaptor proteins from interacting with it.

### Gp53 modulates the activity of the ClpCP protease in B. subtilis

**Figure 4.**
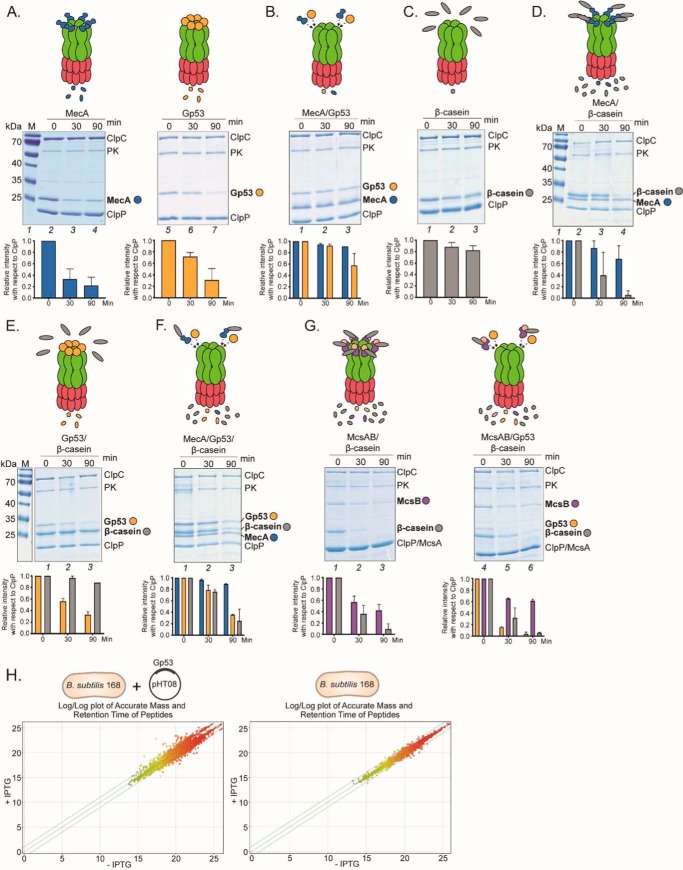
**Gp53 modulates the specificity of the ClpCP protease in *B. subtilis*.** *A*, representative images of SDS-PAGE gels of *in vitro* degradation of MecA and Gp53 by ClpCP protease. The intensities of the bands corresponding to MecA or Gp53 are shown in the graph relative to the intensity of the ClpP band in the corresponding lanes. The migration positions of ClpC (1 μm), MecA (1 μm), Gp53 (1 μm), and ClpP (1 μm) are indicated. Pyruvate kinase (*PK*, 20 ng/ml) and phosphoenolpyruvate (4 mm) were used as an ATP generation system. *B*, as in *A*, but equimolar amounts of MecA and Gp53 were added together. *C*, as in *A*, but the *in vitro* degradation assays were conducted in the presence of 3 μm β-casein and in the absence of MecA or Gp53. *D*, as in *C*, but the *in vitro* degradation assays were conducted in the presence of MecA. *E*, as in *C*, but the *in vitro* degradation assays were conducted in the presence of Gp53. *F*, as in *C*, but the *in vitro* degradation assays were conducted in the presence of MecA and Gp53. *G*, as in *C*, but the *in vitro* degradation assays were conducted with McsA/B (1 μm each) in the absence and presence of Gp53. In *A–G*, the same color coding is used in the schematics, gels, and graphs to aid data interpretation. *H*, *left panel*, a log/log plot comparing the accurate mass and retention time of peptides in whole-cell extracts of *B. subtilis* containing pHT08-Gp53 expressing Gp53 upon induction by IPTG and control whole-cell extracts of *B. subtilis* containing pHT08-Gp53 to which no IPTG was added. A paired *t* test (*p* < 0.05) was carried out to identify peptides that had a -fold change in abundance of 2 or more and that lie on or outside of the *diagonal outer green lines. Right panel*, as in the left graph, but whole cell-extracts of *B. subtilis* with and without IPTG added were compared, which demonstrated that the change in peptide abundance was specific to the presence of Gp53.

Because adaptor proteins like MecA are required for activation and to confer substrate specificity upon the ClpCP protease, we next investigated the effect of Gp53 on the protease activity of ClpCP. Therefore, to determine whether Gp53 inhibits the proteolytic activity of the ClpCP protease or merely modulates its activity during SPO1 development in *B. subtilis*, we conducted *in vitro* protein degradation assays. As shown in Fig. 4*A*, *left panel*, in the absence of any substrate, MecA, as expected (21), was degraded by ClpCP protease. Similarly, Gp53 was also degraded, albeit at a slower rate than MecA, by the ClpCP protease (Fig. 4*A*, *right panel*). Further, consistent with the results in Fig. 3, the results in Fig. 4*B* confirmed that both Gp53 and MecA compete for binding to ClpC because addition of both proteins together resulted in an overall decreased rate of degradation of either protein (compare *lanes 3*, *4*, *6*, and *7* in Fig. 4*A* with *lanes 2* and *3* in Fig. 4*B*).

Next we conducted protein degradation assays using the intrinsically unfolded β-casein as a model substrate in the presence of MecA and/or Gp53. Consistent with previous studies, the control reaction in the absence of MecA or Gp53 did not result in the degradation of β-casein (Fig. 4*C*). However, degradation of β-casein was detected in the presence of MecA (compare *lanes 1–3* in Fig. 4*C* with *lanes 2–4* in Fig. 4*D*). Interestingly, although ClpC is activated by Gp53 (Fig. 3), leading to degradation of Gp53 by ClpCP (Fig. 4*A*), β-casein was not degraded in the presence of Gp53 (Fig. 4*E*). Consistent with the results in Fig. 4*B*, the presence of Gp53 and MecA together in the reaction decreased the rate of β-casein degradation (Fig. 4*F*): following 90 min of incubation, ∼10-fold β-casein was left intact compared with reactions without Gp53 (compare *lane 4* in Fig. 4*D* with *lane 3* in Fig. 4*F*). Interestingly, additional experiments with a different adaptor protein, McsB/A (McsB requires McsA for activation (22)), revealed that Gp53 is less efficient at competing for ClpC in the presence of McsB/A. As shown in Fig. 4*G*, the rate of degradation of β-casein in reactions with McsB/A in the absence or presence of Gp53 was indistinguishable (compare *lanes 4–6* with *lanes 1–3*). Overall, the results clearly indicate that Gp53 does not inhibit the proteolytic activity of ClpCP protease but could compete with some host adaptor proteins to modulate the activity of ClpCP protease during SPO1 development in *B. subtilis*.

To provide evidence of Gp53-mediated modulation of the activity of ClpCP protease on a proteome-wide scale, we compared the proteome profiles of *B. subtilis* cells expressing Gp53 (Fig. 4*H*, *left panel*) with those of cells not expressing Gp53 (Fig. 4*H*, *right panel*) by LC-MS. The log/log plot of the mass-to-retention time of the peptides revealed that the majority of peptides within the *B. subtilis* proteome remained unaffected in the presence of Gp53 (Fig. 4*H*, *left panel*). However, it appeared that a targeted subset of peptides is significantly altered by 2-fold or more specifically because of the presence of Gp53 (Fig. 4*H*, *left panel*). Of the 197 peptides that have altered abundance in Gp53-expressing cells, 79 were sequenced using an auto-MS/MS method and were mapped to 34 unique proteins involved in diverse biological activities (Table S3). This equates to ∼1% of the total known proteome of *B. subtilis* (proteome ID UP000001570, https://www.uniprot.org/proteomes)^4^ (24) and is likely an underestimate because not all peptides were sequenced using this method. Importantly, we note that peptides of proteins shown in Table S3 are not just found with higher abundance (suggesting protection from degradation) but also with lower abundance (suggesting possible accelerated degradation) in the presence of Gp53. In conclusion, the results unambiguously reveal that Gp53 could compete with some bacterial adaptor proteins for binding to the ClpC and therefore does not inhibit but modulates the activity of the ClpCP protease during SPO1 development.

### Compromised ClpCP protease activity affects the efficacy of SPO1 development in B. subtilis

**Figure 5.**
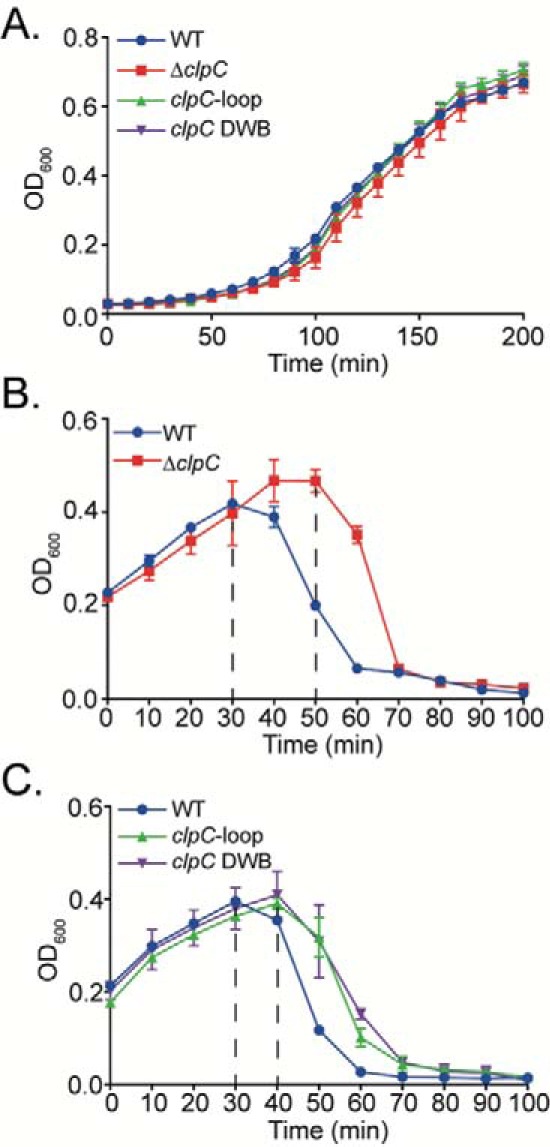
**Compromised ClpCP protease activity affects the efficacy of SPO1 development in *B. subtilis*.** *A*, graph showing the growth curves of WT, Δ*clpC* (IH25), *clpC* DWB (IH140), and *clpC*-loop (IH217) *B. subtilis* cultures. *B*, graph showing the optical density as a function of time of a culture of exponentially growing WT and Δ*clpC B. subtilis* cells following infection with SPO1 at *A*_600_ 0.2. *C*, as in *B*, but with WT, *clpC* DWB, and *clpC*-loop *B. subtilis* cells. *Error bars* in *A–C* represent S.E. (*n* = 3).

We posited that if the role of Gp53 is to modulate the activity of ClpCP protease to allow successful development of SPO1 in *B. subtilis*, then a Δ*clpC B. subtilis* strain (IH25) would provide a compromised host environment for SPO1 development compared with WT *B. subtilis* cells. Thus, we compared the ability of SPO1 to lyse an exponentially growing culture of WT and Δ*clpC B. subtilis* by measuring cell density (light absorbance at *A*_600_) as a function of time following SPO1 infection. The growth of WT and Δ*clpC* strains under our experimental conditions did not detectably differ (Fig. 5*A*). A rapid drop in cell density, indicating cell lysis, was observed after ∼30 min in the WT *B. subtilis* culture infected with SPO1 at *A*_600_ 0.2 (Fig. 5*B*). As expected, the Δ*clpC B. subtilis* culture infected with SPO1 continued to grow for a further 20 min, reaching a higher cell density than the WT strain before undergoing cell lysis (Fig. 5*B*). As shown in Fig. 5*C*, similar results were obtained with *B. subtilis* strains containing ClpC, which is unable to hydrolyze ATP because of two mutations within the Walker B domain in both ATPase domains (*clpC* DWB, strain IH140 (18)) or unable interact with ClpP because of a deletion in a region required for binding to ClpP (*clpC*-loop VGF::GGR, strain IH217 (25)). In the case of the *clpC* DWB and *clpC*-loop mutant *B. subtilis* strains, the culture continued to grow for a further 10 min compared with the WT culture before cell lysis occurred. Overall, the results are consistent with the findings above and indicate that modulating the activity of ClpCP protease by Gp53, but not its inhibition, is required for optimal SPO1 development in *B. subtilis*.

## Discussion

A common theme by which phages affect host physiology to benefit phage progeny development is through the modulation or inhibition of bacterial cellular processes (1, 2). Previous studies (6–9) revealed that SPO1 infection results in remodeling of several host processes by seven (Gp38, Gp39, Gp40, Gp44, Gp50, Gp51, and Gp56) of the 26 genes encoded by the host takeover module. Specifically, although the molecular details still remain elusive, Gp38, Gp39, Gp40, Gp44, Gp50, and Gp51 have been implicated in shutoff of host macromolecular biosynthetic processes (RNA, DNA, and protein synthesis) and Gp56 in inhibition of bacterial cell division (6–8). This study revealed that Gp37, Gp41, Gp42, Gp44, Gp46, Gp53, Gp56, and Gp60 attenuate the growth of *B. subtilis* in the absence of SPO1 infection (Fig. 1). It seems that the individual effects of some host takeover module gene products (*e.g.* Gp38, Gp39, and Gp40) might not be sufficient to affect bacterial growth. In support of this view, coexpression of Gp38, Gp39, and Gp40, which constitute operon 2 of the host takeover module (Fig. 1*A*), resulted in increased growth attenuation, presumably through synergistic activities of Gp38, Gp39, and Gp40. As phage genomes tend to be compact and efficient, it is remarkable that SPO1 has evolved many elaborate mechanisms to take over *B. subtilis* cells. We predict that the action of each individual host takeover module gene product is carefully regulated in a temporally coordinated manner and that some functionally interact with each other to bring about the desired effect (*e.g.* Gp38, Gp39, and Gp40) or control their functionalities. The observation that coexpression of Gp45 with Gp46 (operon 3) counteracts the effect of the latter on *B. subtilis* growth (Fig. 1*E*) further underscores this view. Further, it is tempting to speculate that genes within operon 3 of the host takeover module are akin to a toxin/anti-toxin module. Previous investigations by Stewart and co-workers (6–9) have shown that mutations in genes 38, 39, 40, 44, 50, 51, and 56 do not decrease the burst size of SPO1 infection. It is important to remember that most studies of phage–host interactions, like the present one and those listed above, are conducted under “optimal” laboratory conditions. Thus, it is possible that some of the SPO1 host takeover module gene products might only be required for infecting and replicating in bacteria in different physiological states, *e.g.* a nutrient-starved population of bacteria (26). For example, Gray *et al.* (27) reported recently that *B. subtilis* can exist in an oligotrophic state without sporulating. It would thus be interesting to investigate whether some SPO1 host takeover gene products and their targets become essential for SPO1 development in *B. subtilis* experiencing oligotrophic growth conditions. Further, our earlier work on the T7 phage led to the identification of a T7 gene product involved in inhibition of the bacterial RNA polymerase only in the stationary phase of growth (28).

The involvement of bacterial protein degradation machinery in phage development is not uncommon, and well established examples include lysis–lysogeny decision in phage λ (29), DNA replication/transcription decision in phage μ (30), and inhibition of Lon protease by T4 (31). Thus, it seems that Gp53 is one of a growing number of phage-encoded factors that are involved in modulating the activity of host protein degradation machinery optimal phage progeny development.

Under standard laboratory conditions, the absence of ClpC had a subtle yet consistent detrimental effect on the efficacy of SPO1 infection in *B. subtilis* (Fig. 5). Thus, it is possible that the requirement for ClpC by SPO1 becomes more prominent under more native and/or specific conditions for *B. subtilis* (see above). The results reveal that SPO1 Gp53 competes with some host adaptor protein(s) for binding to ClpC and thereby modulates the activity of the ClpCP protease. Because different adaptor proteins can compete for binding to ClpC to confer substrate specificity upon the ClpCP protease (14, 17), it seems that Gp53 can be considered an adaptor-like protein produced by a phage. Consistent with this view, the results revealed that the binding site of Gp53 on ClpC is likely to overlap with that of native adaptor proteins such as MecA or McsB (Fig. 3*E*), and, like native adaptor proteins, Gp53 becomes degraded by the ClpCP protease in the absence of any substrates (Fig. 4*B*). Thus, it is conceivable that Gp53 functionally mimics the role of a *B. subtilis* adaptor protein, which, consequently, could result in subversion of the ClpCP protease to benefit phage development. However, the amino acid sequences of *B. subtilis* adaptor proteins and Gp53 share very little sequence similarity (Fig. S3). In conclusion, we propose the following two mutually exclusive scenarios: (1) Gp53 can act like an adaptor-like protein, target SPO1-derived substrates for proteolysis, and, consequently, interferes with the recognition and targeting of bacterial substrates by native (bacterial) adaptor proteins for proteolysis by the ClpCP protease, and/or (2) Gp53 repurposes the ClpCP protease to modulate the proteome of *B. subtilis* to benefit SPO1 development. The fact that the ClpCP protease and its adaptor proteins are involved in both regulatory (*e.g.* transcription factors) and general (misfolded or damaged proteins) proteolysis (32), it would seem that that a competing “xenogeneic” adaptor-like protein such as Gp53 would have detrimental pleiotropic effects on the growth of *B. subtilis* cells (Fig. 1). Future work in the laboratory will be directed at experimentally investigating these scenarios. Finally, we note that virus-directed degradation of host proteins is not uncommon in eukaryotic systems. For example, in the case of human papillomaviruses, the virus-encoded E6 and E6-AP proteins interact with the cell cycle control protein p53 and target it for degradation, increasing the oncogenic potential of human papillomaviruses (33).

The ClpC and ClpP proteins of Gram-negative and Gram-positive bacteria have recently been recognized as viable targets for antibiotic discovery, and a number of naturally occurring antibacterial products deregulate the respective activities of ClpC or ClpP, resulting in bacterial cell death (34, 35). With the emerging interest in the use of phages and phage-encoded proteins as sources of alternatives to antibiotics, this study reveals that the ClpCP protease of *B. subtilis* and homologs in other bacteria can be subjected to xenogeneic dysregulation by phage-derived factors and adds Gp53 to the growing list of naturally occurring antibacterial products that target the bacterial protein degradation machinery.

## Materials and methods

### Plasmids, strains, and proteins

All plasmids used in this study for protein expression and BTH assays were generated using standard molecular biology procedures and are detailed in Table S1. pSCBAD-Gp53 was made by Gibson assembly (36). The pSC101 plasmid (37) was modified by inserting the regulatory region of pBAD33 (*araC* promoter region, multiple cloning sites, and the rrnB T2 terminator) between restriction sites XhoI and NsiI. All proteins used in this study were purified by either Ni affinity chromatography (for His_6_-tagged proteins, *i.e.* Gp53, MecA, and ClpP) or anti-FLAG M2 affinity resin (for FLAG-tagged proteins *i.e.* ClpC) using standard molecular biology procedures. The details of plasmids used for protein purification are shown in Table S1. All the strains used in this study are shown in Table S2.

### Bacterial growth assays

Unless otherwise stated, *B. subtilis* cultures were grown in 2xYT (16 g/liter tryptone, 10 g/liter yeast extract, 5.0 g/liter NaCl) medium (Sigma) with 2% (w/v) glucose and appropriate antibiotics at 37 °C. For the experiments shown in Figs. 1 and 2*A*, seed cultures were grown at 37 °C with shaking at 700 rpm for 16–18 h in a THERMOstar (BMG Labtech) plate incubator by directly inoculating a colony into 200 μl of 2xYT medium containing 5 μg/ml chloramphenicol and 2% (w/v) glucose (to prevent leaky expression from the pHT01 vector) into a 48-well plate (Greiner). The growth curves were also performed in 48-well plates in a SPECTROstar Nano Absorbance multiwell plate reader (BMG Labtech). The seed cultures were *A*_600_-corrected to 0.025 in 200 μl of fresh 2xYT medium containing 5 μg/ml chloramphenicol, 2% (w/v) glucose, and either water or 1 mm IPTG to induce the expression of SPO1 host takeover genes. Cultures were incubated at 37 °C with shaking at 700 rpm. At least three biological and technical replicates were performed.

### Pulldown assays

These were performed as described previously (28) using proteins specified in the main text and figures, with the following amendments. Binding buffer (25 mm NaH_2_PO_4_, 50 mm NaCl, 5 mm imidazole, and 5% glycerol (pH 7)), wash buffer (25 mm NaH_2_PO_4_, 50 mm NaCl, 15 mm imidazole, and 5% glycerol (pH 7)), and samples were eluted by adding 50 μl of Laemmli 2× concentrated SDS sample buffer to beads and boiled for 5 min prior to analysis by SDS-PAGE.

### Bacterial two-hybrid interaction assays

These were carried out using the bacterial adenylate cyclase-based two-hybrid system (Euromedex) and conducted according to the manufacturer's guidelines. Briefly, recombinant plasmids encoding proteins of interest fused to the T25 or T18 domain of adenylate cyclase were transformed into competent DHM1 cells (see Table S1 for details of plasmids used). Transformants were grown overnight at 30 °C in a 96-well plate in LB medium containing ampicillin (100 μg/ml), kanamycin (50 μg/ml), and IPTG (0.5 mm). Each culture was then diluted 1:5 in Z buffer (45 mm Na_2_HPO_4_–NaH_2_PO_4_ (pH 7), 10 mm KCl, 2 mm MgSO_4_·7H_2_O, and 40 mm β-mercaptoethanol), and cells were permeabilized using 0.01% (w/v) SDS and 10% (v/v) chloroform. Each culture was again diluted 1:4 in Z buffer and equilibrated at 28 °C before adding 0.4% (v/v) *O*-nitrophenol-β-galactoside. Reactions were carried out in a SPECTROstar Nano Absorbance multiwell plate reader (BMG Labtech) at 28 °C for 20 min, with measurement of *A*_420 nm_ every 1 min. The β-gal activity is given in Miller units, with 1 Miller unit corresponding to 1 nm *O*-nitrophenol-β-galactoside hydrolyzed per minute at 28 °C (after accounting for *A*_600_ correction and dilution factors). At least three biological and technical replicates were performed for each measurement.

### ATPase assays

The ATPase assay is based on colorimetric measurement of the concentration of P_i_ from the hydrolysis of ATP. Reactions were carried out at 37 °C for the specified times in buffer containing 100 mm KCl, 25 mm Tris-HCl (pH 8.0), 5 mm MgCl_2_, 0.5 mm DTT, 0.1 mm EDTA, 0.5 μg/μl BSA, and 4 mm ATP. ClpC, MecA, and/or Gp53 were added at concentrations indicated in the figures and figure legends. The amount of P_i_ in the reaction was then quantified using PiColorLock^TM^ detection reagent (Innova Biosciences) according to the manufacturer's guidelines. The data were corrected for buffer-only values to account for any spontaneous degradation of ATP. At least three biological and technical replicates were performed for each reaction.

### ClpCP-mediated protein degradation assays

These were conducted exactly as described previously (15). The protein components were present in the amounts indicated in the figure legends.

### LC-MS

Six biological replicates of *B. subtilis* containing pHT08 encoding His-Gp53 were grown to *A*_600_ 0.5. Three replicates were induced with 1 mm IPTG, and water was added to the remaining three as uninduced controls. Cultures were left to grow for 2 h and corrected to *A*_600_, and 2 and 5 ml were pelleted at 3220 × *g* for 10 min. Proteins were precipitated from the pellets by methanol/chloroform extraction as described previously (23). The dried protein pellets were resuspended in 100 μl of 0.1 m ammonium bicarbonate (AmBic)–10% methanol. 125 mm DTT in 0.1 m AmBic–10% methanol was added and incubated at 80 °C for 15 min. 250 mm 2-iodoacetamide in 0.1 m AmBic–10% methanol was added and incubated at room temperature in the dark for 30 min. Trypsin at 0.11 μg/μl was added, and samples were incubated for 16 h at 37 °C. All samples were injected in a randomized order and separated on a 1290 LC system (Agilent) operating in normal flow mode at 200 μl/min. 1 μl of the sample was separated on a Zorbax Extend-C18 column (1.8 μm particle size, 5 cm length, 2.1 mm identity (Agilent)). A 19.5-min method with the following gradient was used: 97% buffer A (0.1% (v/v) formic acid in water), 3% buffer B (0.1% (v/v) formic acid in acetonitrile). Buffer B was increased to 40% over 12.5 min, followed by an increase to 100% buffer B over 2 min, where it was held for 2 min. Buffer B was then ramped back down to 3% over 1 min and equilibrated for 2 min prior to the next injection. The 1290 LC system was coupled to a 6550 iFunnel Q-ToF mass spectrometer (Agilent) equipped with an iFunnel electrospray source and running MassHunter data acquisition software. For relative peptide abundance, data were acquired in MS-only mode over the 300–1700 *m*/*z* range at the rate of 1 spectrum/s. Peptides were identified using an auto-MS/MS method selecting up to 20 precursors per cycle with a threshold of 5000 counts and a charge state of 2 or greater. Profinder B08 (Agilent) was used for peak list generation. The resulting MS-only files were imported into Mass Profiler Professional (Agilent) for relative peptide abundance comparison. Samples grouped according to type and features (as defined by accurate mass and retention time) were clustered for further analysis. Results were filtered so that a feature was present in all samples within both groups. Log/log of the intensity of features in each sample group was plotted, and a paired *t* test (*p* < 0.05) was carried out to identify features that had a -fold change of 2 or higher. LC-MS/MS data were extracted into peak lists using SpectrumMill (Agilent). Database searching against SwissProt was achieved using the following search parameters: enzyme specificity, trypsin; charge states, 2 or greater; fixed modification, cysteine carbaminoacetylation; variable modifications, methionine-oxidized. A score of 5 was set for acceptance of peptide assignments and 10 for protein identifications. Peptides with a false discovery rate of more than 5% were discarded.

### SPO1 infection assays

Seed cultures of bacteria were grown at 37 °C with shaking at 700 rpm for 16–18 h in a THERMOstar (BMG Labtech) plate incubator by directly inoculating a colony into 1 ml of 2xYT medium into a 24-well plate (Greiner). The infection curves were also performed in 24-well plates in a SPECTROstar Nano Absorbance multiwell plate reader (BMG Labtech). The seed cultures were *A*_600_-corrected to 0.05 in 1 ml of fresh 2xYT medium and incubated at 37 °C with shaking at 700 rpm. At *A*_600_ 0.2, a certain amount of SPO1 lysate was added in a 1:1 ratio of bacterial cells; phage particles and *A*_600_ measurements were taken every 10 min until full lysis of the bacterial culture occurred. At least three biological and technical replicates were performed.

## Author contributions

N. M., I. H., L. B., S. N., D. B., K. T., and S. W. investigation; N. M., I. H., K. T., and S. W. writing-review and editing; I. H. and S. W. methodology; K. T. and S. W. supervision; K. T. and S. W. writing-original draft; S. W. conceptualization; S. W. formal analysis; S. W. funding acquisition; S. W. validation; S. W. visualization; S. W. project administration.

## Supplementary Material

Supporting Information

## Footnotes

4 Please note that the JBC is not responsible for the long-term archiving and maintenance of this site or any other third party–hosted site.

## Footnotes

3 The abbreviations used are:

IPTG: isopropyl 1-thio-β-d-galactopyranoside
BTH: bacterial two-hybrid
AmBic: ammonium bicarbonate
ANOVA: analysis of variance
DWB: double walker B.
