# Supplementary material for: Xenogeneic modulation of the ClpCP protease of Bacillus subtilis by a phage-encoded adaptor-like protein
Source: J Biol Chem. 2019 Jul 30;294(46):17501–11. doi: 10.1074/jbc.RA119.010007 (PMC6873191; doi:10.1074/jbc.RA119.010007)
Supplement: Supporting Information [file supp_294_46_17501__index.html]

Xenogeneic modulation of the ClpCP protease of Bacillus subtilis by a phage-encoded adaptor-like protein — Xenogeneic modulation of the ClpCP protease — Xenogeneic modulation of the ClpCP protease of Bacillus subtilis by a phage-encoded adaptor-like protein — Xenogeneic modulation of the ClpCP protease — Supporting Information 

# Xenogeneic modulation of the ClpCP protease of *Bacillus subtilis* by a phage-encoded adaptor-like protein

## Supporting Information

- Supporting Information (to be published online) - Supporting Information
