# Supplementary material for: Xenogeneic modulation of the ClpCP protease of Bacillus subtilis by a phage-encoded adaptor-like protein
Source: J Biol Chem. 2019 Jul 30;294(46):17501–11. doi: 10.1074/jbc.RA119.010007 (PMC6873191; doi:10.1074/jbc.RA119.010007)
Supplement: Supporting Information [file supp_RA119.010007_154011_1_supp_368372_pv5v5h.pdf]

Xenogeneic regulation of the ClpCP protease of *Bacillus subtilis* by a phage-encoded adaptor-like protein

Nancy Mulvenna[1], Ingo Hantke[2], Lynn Burchell[1], Sophie Nicod[1], David Bell[3], Kürşad Turgay[2] and Sivaramesh Wigneshweraraj[1]*

[1]MRC Centre for Molecular Bacteriology and Infection, Imperial College London, London, SW7 2AZ, UK; [2]Institute für Mikrobiologie, Leibniz Universität Hannover, Herrenhäuser Str. 2, 30419 Hannover, Germany. [3] SynbiCITE, iHub, Imperial College London, White City, London W12 0BZ, UK. [4]Max Planck Unit for the Science of Pathogens, Chariteplatz 1, 10117 Berlin, Germany

Figure S1

Figure S2

Figure S3

Table S1

Table S2

Table S3

Figure S1

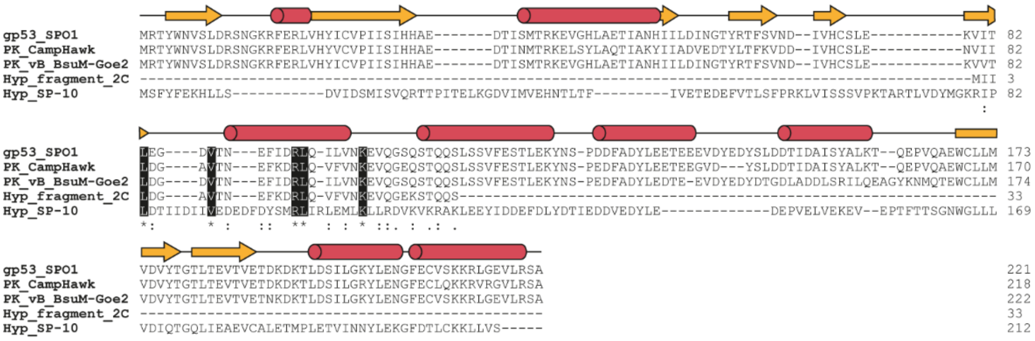

Fig. S1. Alignment of amino acid sequences from Gp53-like proteins from *B. subtilis* phages. The localization of the β-strands and α-helices in Gp53 are indicated by yellow arrows and red cylinders, respectively. Residues which are conserved across all sequences that were targeted for mutagenesis are highlighted in black.

Figure S2

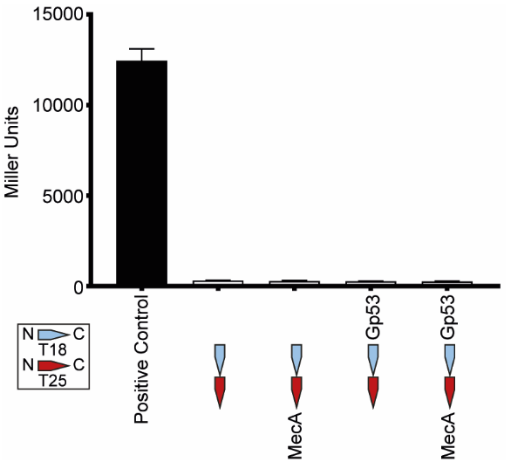

Fig. S2. Bar chart showing the results from the bacterial two hybrid assay of Gp53 with MecA. Error bars represent SEM (n=3).

Figure S3

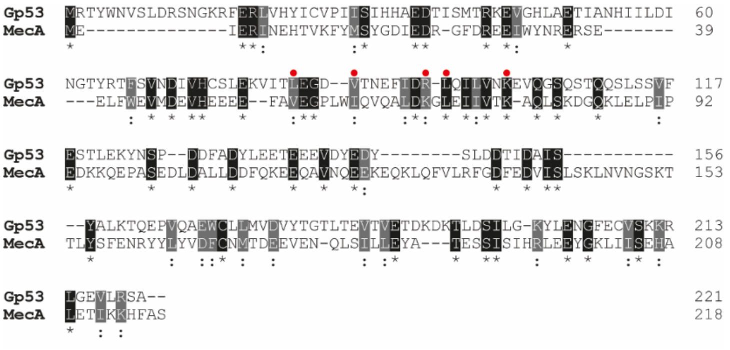

Fig. S3. Alignment of amino acid sequences from *Bacillus subtilis* MecA and SPO1 Gp53. Conserved residues are highlighted in black.

Table S1

Plasmids used during this study

Plasmid Name	Marker	Inducer used	Application	Ref
pHT01	Amp^R *E. coli* Cam^R *B. subtilis*	IPTG	Cloning	2BScientific
pHT08	Amp^R *E. coli* Cam^R *B. subtilis*	IPTG	Cloning	2BScientific
pHT01-GFP	Amp^R *E. coli* Cam^R *B. subtilis*	IPTG	Growth attenuation assays	This study
pHT01-Gp37	Amp^R *E. coli* Cam^R *B. subtilis*	IPTG	Growth attenuation assays	This study
pHT01-Gp38	Amp^R *E. coli* Cam^R *B. subtilis*	IPTG	Growth attenuation assays	This study
pHT01-Gp39	Amp^R *E. coli* Cam^R *B. subtilis*	IPTG	Growth attenuation assays	This study
pHT01-Gp40	Amp^R *E. coli* Cam^R *B. subtilis*	IPTG	Growth attenuation assays	This study
pHT01-Gp41	Amp^R *E. coli* Cam^R *B. subtilis*	IPTG	Growth attenuation assays	This study
pHT01-Gp42	Amp^R *E. coli* Cam^R *B. subtilis*	IPTG	Growth attenuation assays	This study
pHT01-Gp43	Amp^R *E. coli* Cam^R *B. subtilis*	IPTG	Growth attenuation assays	This study
pHT01-Gp44	Amp^R *E. coli* Cam^R *B. subtilis*	IPTG	Growth attenuation assays	This study
pHT01-Gp45	Amp^R *E. coli* Cam^R *B. subtilis*	IPTG	Growth attenuation assays	This study
pHT01-Gp46	Amp^R *E. coli* Cam^R *B. subtilis*	IPTG	Growth attenuation assays	This study
pHT01-Gp47	Amp^R *E. coli* Cam^R *B. subtilis*	IPTG	Growth attenuation assays	This study
pHT01-Gp48	Amp^R *E. coli* Cam^R *B. subtilis*	IPTG	Growth attenuation assays	This study
pHT01-Gp49	Amp^R *E. coli* Cam^R *B. subtilis*	IPTG	Growth attenuation assays	This study
pHT01-Gp50	Amp^R *E. coli* Cam^R *B. subtilis*	IPTG	Growth attenuation assays	This study
pHT01-Gp51	Amp^R *E. coli* Cam^R *B. subtilis*	IPTG	Growth attenuation assays	This study
pHT01-Gp52	Amp^R *E. coli* Cam^R *B. subtilis*	IPTG	Growth attenuation assays	This study
pHT01-Gp52.1	Amp^R *E. coli* Cam^R *B. subtilis*	IPTG	Growth attenuation assays	This study
pHT01-Gp52.2	Amp^R *E. coli* Cam^R *B. subtilis*	IPTG	Growth attenuation assays	This study
pHT01-Gp53	Amp^R *E. coli* Cam^R *B. subtilis*	IPTG	Growth attenuation assays	This study
pHT01-Gp54	Amp^R *E. coli* Cam^R *B. subtilis*	IPTG	Growth attenuation assays	This study
pHT01-Gp55	Amp^R *E. coli*	IPTG	Growth attenuation	This study

	Cam^R *B. subtilis*		assays	
pHT01-Gp56	Amp^R *E. coli* Cam^R *B. subtilis*	IPTG	Growth attenuation assays	This study
pHT01-Gp57	Amp^R *E. coli* Cam^R *B. subtilis*	IPTG	Growth attenuation assays	This study
pHT01-Gp58	Amp^R *E. coli* Cam^R *B. subtilis*	IPTG	Growth attenuation assays	This study
pHT01-Gp59	Amp^R *E. coli* Cam^R *B. subtilis*	IPTG	Growth attenuation assays	This study
pHT01-Gp60	Amp^R *E. coli* Cam^R *B. subtilis*	IPTG	Growth attenuation assays	This study
pHT01-Operon1	Amp^R *E. coli* Cam^R *B. subtilis*	IPTG	Growth attenuation assays	This study
pHT01-Operon2	Amp^R *E. coli* Cam^R *B. subtilis*	IPTG	Growth attenuation assays	This study
pHT01-Operon3	Amp^R *E. coli* Cam^R *B. subtilis*	IPTG	Growth attenuation assays	This study
pHT01-Operon4	Amp^R *E. coli* Cam^R *B. subtilis*	IPTG	Growth attenuation assays	This study
pHT01-Operon5	Amp^R *E. coli* Cam^R *B. subtilis*	IPTG	Growth attenuation assays	This study
pHT01-Operon6	Amp^R *E. coli* Cam^R *B. subtilis*	IPTG	Growth attenuation assays	This study
pHT01-Operon7	Amp^R *E. coli* Cam^R *B. subtilis*	IPTG	Growth attenuation assays	This study
pHT01-Operon8	Amp^R *E. coli* Cam^R *B. subtilis*	IPTG	Growth attenuation assays	This study
pHT08-Gp53	Amp^R *E. coli* Cam^R *B. subtilis*	IPTG	Growth attenuation assays	This study
$pET33b^+$	Kan^R	IPTG	Cloning	Novagen
$pET33b^+$-Gp53	Kan^R	IPTG	Protein purification	This study
$pET33b^+$-MecA	Kan^R	IPTG	Protein purification	This study
$pET33b^+$-ClpP	Kan^R	IPTG	Protein purification	This study
pT7FLAG	Amp^R	IPTG	Cloning	Sigma
pT7FLAG-ClpC	Amp^R	IPTG	Protein purification	This study
pUT18	Amp^R	IPTG	Cloning	Euromedex
pKT25	Amp^R	IPTG	Cloning	Euromedex
pUT18-Gp53	Amp^R	IPTG	BTH assays	This study
pUT18-Gp53 L83A	Amp^R	IPTG	BTH assays	This study
pUT18-Gp53 V87A	Amp^R	IPTG	BTH assays	This study
pUT18-Gp53 R94A	Amp^R	IPTG	BTH assays	This study
pUT18-Gp53 R94E	Amp^R	IPTG	BTH assays	This study
pUT18-Gp53 L95A	Amp^R	IPTG	BTH assays	This study
pUT18-Gp53 K101A	Amp^R	IPTG	BTH assays	This study
pUT18-Gp53 K101E	Amp^R	IPTG	BTH assays	This study
pKT25-ClpC	Kan^R	IPTG	BTH assays	This study

pKT25-ClpC Q18R	Kan^R	IPTG	BTH assays	This study
pKT25-ClpC Q18H	Kan^R	IPTG	BTH assays	This study
pKT25-ClpC L22S	Kan^R	IPTG	BTH assays	This study
pKT25-ClpC H79A	Kan^R	IPTG	BTH assays	This study
pKT25-ClpC NTD	Kan^R	IPTG	BTH assays	This study
pKT25-ClpC NTD D1	Kan^R	IPTG	BTH assays	This study
pKT25-ClpC ΔD2	Kan^R	IPTG	BTH assays	This study
pKT25-ClpC ΔNTD	Kan^R	IPTG	BTH assays	This study
pKT25-ClpC Linker D2	Kan^R	IPTG	BTH assays	This study
pKT25-ClpC D2	Kan^R	IPTG	BTH assays	This study
pUT18-MecA	Amp^R	IPTG	BTH assays	This study
pSC101	Tet^R	N/A	Cloning	ATCC
pBAD33	Amp^R	Arabinose	Cloning	ATCC
pSCBAD-Gp53	Tet^R	Arabinose	Modified BTH assay	This study

Table S2

Strains used in this study.

Strain	Marker	Ref
Escherichia coli		
XL1 blue	N/A	Stratagene
BL21 (DE3)	N/A	Stratagene
DHM1	N/A	Euromedex
Bacillus subtilis		
168 *trpC2*	N/A	(1)
IH25: 168 *trpC2 clpC*::*tet*^{R} *amyE*::P_{veg} *mecA*$_{CTD}$-*gfp* *cam*^{R}	Tet^{R}/Cam^{R}	Provided by Ingo Hantke, derived from (2)
IH140: 168 *trpC2 clpC* E280A E618A (double walker B mutant)	N/A	(3)
IH217: 168 *trpC2 clpC* VGF::GGR (loop mutant)	N/A	(4)

1. Zeigler DR, et al. (2008) The origins of 168, W23, and other Bacillus subtilis legacy strains. *J Bacteriol* 190(21):6983–95.
2. Pan Q, Garsin DA, Losick R (2001) Self-Reinforcing Activation of a Cell-Specific Transcription Factor by Proteolysis of an Anti-σ Factor in B. subtilis. *Mol Cell* 8(4):873–883.
3. Kirstein J, et al. Adaptor protein controlled oligomerization activates the AAA þ protein ClpC. doi:10.1038/sj.emboj.7601042.
4. Molière NP (2012) The role of Bacillus subtilis Clp/Hsp100 proteases in the regulation of swimming motility and stress response. Dissertation (Freie Universität Berlin).

Table S3

Table of the 34 distinct proteins identified by auto LC-MS/MS

Compound	Fold Change
10 kDa chaperonin	2.1
4-hydroxy-3-methylbut-2-enyl diphosphate reductase	2.3
5-epi-aristolochene synthase 3	2.1
60 kDa chaperonin	2.2
60 kDa chaperonin 1	2.1
60 kDa chaperonin 2	2.1
Chaperone protein DnaK	3.5
Chromosomal replication initiator protein DnaA (Fragment)	2.0
Citrate synthase 2	3.3
ComG operon protein 1	2.5
DEAD-box ATP-dependent RNA helicase CshA	3.3
DNA-entry nuclease inhibitor	3.3
Exocyst complex component exo84	2.4
HTH-type transcriptional regulator TauR	8.2
Isoleucine--tRNA ligase	3.0
Lactose operon repressor	3.8
Methylmalonate semialdehyde dehydrogenase [acylating] 1	2.1
Procollagen-lysine,2-oxoglutarate 5-dioxygenase 1	2.6
Protein RecA	3.8
Pyruvate dehydrogenase E1 component subunit alpha	2.1
Ribosome-binding ATPase YchF	7.3
Single-stranded DNA-binding protein A	2.2
Succinate--CoA ligase [ADP-forming] subunit alpha	2.1
Succinate--CoA ligase [ADP-forming] subunit beta	2.4
Uncharacterized protein YqjE	2.2
UPF0738 protein YjbL	2.1
30S ribosomal protein S11	-2.5
Antilisterial bacteriocin subtilosin biosynthesis protein AlbA	-2.2
Flagellin	-2.0
Imidazolonepropionase	-8.6
Isochorismate synthase DhbC	-2.2
Low-temperature-induced 65 kDa protein	-2.0
Oligoendopeptidase F homolog	-2.2
Putative sensory transducer protein YfmS	-3.4
